# Overlapping social structures behind Brazil’s cesarean section births: A decomposition analysis

**DOI:** 10.1371/journal.pone.0325251

**Published:** 2025-06-25

**Authors:** Surbhi Shrivastava, Heeju Sohn

**Affiliations:** Department of Sociology, Emory University, Atlanta, Georgia, United States of America; Ankara University Faculty of Nursing, TÜRKIYE

## Abstract

Increasing cesarean section (CS) rates worldwide have prompted concern for women’s access to quality care and calls for interventions to reduce unnecessary and risky CS. Brazil, where CS births outnumber vaginal births, has one of the highest CS rates in the world. Brazil is also a large and diverse nation, and CS rates differ widely between race/ethnic groups, social classes, and geographic regions. Residential segregation by race/ethnicity and their associations with social class complicate the picture of how each contributes to CS rates and disparities. This article untangles the intersecting social and contextual factors to identify opportunities for interventions to reduce overall CS rates as well as disparities in Brazil. Using Brazil’s national birth registry data from 2019 (n = 2,567,039), this article quantifies how much socioeconomic, prenatal care, pregnancy risk, and geographic factors contribute to racial and ethnic disparities in CS. We applied the Karlson-Holm-Breen (KHB) decomposition method to multivariate logistic regression models. Our findings show that women’s individual risk factors—educational attainment, social status, age, prenatal care, and pregnancy profile—were significant contributors but did not entirely explain racial and ethnic disparities in CS. Geographic factors—where race/ethnic groups tended to live and the region’s risk for CS—also emerged as strong correlates of CS and partially explained unequal rates. The findings untangle the overlapping social structures that predispose some race and ethnic groups to a greater risk of CS and increase overall CS prevalence in Brazil.

## Introduction

Large racial and ethnic differences in cesarean sections (CS) persist in Brazil, where CS rates far exceed recommended levels [1,2]. More than half of births in Brazil were via CS in 2018 [3], with white women reporting the highest proportion of CS births [4,5]. While CS is a life-saving procedure, it is not without risk; both the mother and the infant face short- and long-term complications from CS [6,7]. Global research considers population CS rates of 5–10% optimal, and rates above 15% do more harm than good [8]. Scholars have described birth care in Brazil as “aggressive management” with excessive interventions [9], shifting CS births from necessary exceptions to routine procedures [10]. Excess CS rates often result from care that is not based on medical evidence [11], and researchers point to social factors as crucial determinants of high CS rates [12,13]. Although CS prevalence varies widely between race and ethnic groups in Brazil, less is known about how different factors contribute to this disparity.

Whereas CS rates in Brazil have been a subject of study for decades, a relatively small body of research has examined the paradoxical racial and ethnic disparities in CS. Women from marginalized racial and ethnic groups have poorer maternal health outcomes overall [14], but CS rates show an opposite pattern. Maternal mortality rates are 2.5 times higher for Black women than for white women [15], and Black and Brown women receive less prenatal care and experience more pregnancy complications during and after birth [16]. Yet, White women who, on average, have greater socioeconomic advantages and access to care are more likely to give birth via CS [4]. This relationship between CS and maternal health is unlike the pattern observed in the United States – another large racialized nation – where high CS rates are often reflections of poor prenatal care and biases in healthcare settings [17,18]. Instead, the higher rate of CS among white women in Brazil is similar to patterns in lower-income countries such as Sudan, Uganda, and India, where women with greater social status have better access to medical interventions [19–21].

A variety of factors can contribute to the observed racial and ethnic CS disparity in Brazil. Demographic shifts over time may affect Brazil’s higher CS rates among White women. As Brazil’s population fertility rates declined over the past 50 years, the ages at which women become mothers have increased the fastest among White women, especially those living in urban areas and those with more education [22]. Pregnancy complications that may require CS deliveries increase with the mother’s age [23], and the unequal pace of fertility transition may have contributed to racial and ethnic gaps in CS rates. Black and brown women still experience higher parity and smaller intervals between pregnancies compared to white women in Brazil [14]. However, controlling for pregnancy risk factors such as maternal age and parity does not account for the wide CS disparity between racial and ethnic groups.

Racial and ethnic differences in CS may also be a by-product of geographic differences in CS prevalence within Brazil. The country’s universal health system, *Sistema Única de Saúde (*SUS), operates in a decentralized model where localities assume responsibility for co-financing health programs and delivering services [24–26]. Pregnant women accessing SUS receive prenatal care from neighborhood-based primary physicians and are referred to a nearby tertiary hospital for obstetrics and gynecology care, including childbirth. Under the SUS model, women’s healthcare environment and access to care are determined by where they live [27,28], and racial and ethnic residential patterns can produce disparities in CS rates between groups.

The interplay of sociocultural factors and healthcare delivery in Brazil can also shape racial and ethnic disparities in CS births. Studies frequently cite maternal preference as the reason behind high CS births in Brazil. CS may appeal to some women with resources because medical interventions may be interpreted as better quality care [29]. Others may seek CS to avoid the pain associated with vaginal birth, to maintain sexual performance, and to time the delivery [10,30]. The type of health facility can influence maternal preference; private hospitals have much higher rates of CS than public ones. Furthermore, women’s perceptions of CS are influenced by their social context, including their educational attainment, norms, and expectations in their social networks, and histories of interactions with healthcare providers [31]. Ethnographic and qualitative data document how racism operates in healthcare settings and predisposes pregnant women to stigma, disrespect, and poor quality care [15]. Women who don’t identify as white may also experience higher rejection from the first tertiary hospital they turn to for birth care [14]. Thus, women’s experiences of racial and ethnic discrimination during pregnancy and birth suggest that unjust structural processes may produce unequal birth outcomes by race and ethnicity [15]. To what extent these factors contribute to differential rates of CS among Brazilian racial and ethnic groups is unclear.

The relative lack of research extricating racial and ethnic disparities in CS rates is indicative of insufficient scientific inquiry into broader ‘race and health’ issues in Brazil [32]. Scholars argue for concerted efforts to study the nature of racial and ethnic health disparities owing to Brazil’s long history of indigenous oppression, transatlantic slave trade, and miscegenation between racial groups that result in unique race relations. Unlike the United State’s ancestry-oriented models of race and ethnicity [33], the Brazilian model reflects a continuum of skin color where multiple shades of brown exist between white and Black as endpoints [34,35]. As such, many Brazilians prefer the racially ambiguous term *moreno* (Brown) over the racial affirmation attached to *negro* (Black) [36]. Measurements of race and ethnicity in Brazil rely on a skin color continuum and have often relied on predetermined categories through self-classification and observation [37]. In recent decades, affirmative action policies and growing Black consciousness have contributed to more people identifying with darker skin colors [35]. Yet, a prominent section of the discourse on race relations in Brazil projects images of racial harmony and privileges socioeconomic differences over racial and ethnic inequality as the primary cause for health disparities [38,39].

This article fills a gap in the public health discourse surrounding CS rates in Brazil by quantifying the degree to which potential confounders explain the racial and ethnic disparities in CS rates. Specifically, this article addresses the following research question: *How much do sociodemographic differences, access to prenatal care, disparity in pregnancy risk factors, and unequal distribution of women across geographies explain the racial/ethnic gaps in CS rates in Brazil?*

Using the 2019 national registry of births data, the year before the COVID-19 pandemic, we quantified how much differences in sociodemographic, pregnancy-related risk, and geographic factors contributed to the racial and ethnic disparities in CS rates. Formal decomposition analyses showed that underlying sociodemographic and geographic differences between women of different race and ethnic groups were significant correlates but did not entirely explain the disparities. The findings refocus on the social contexts that predispose some race and ethnic groups to a higher CS risk and call upon programs to incorporate Brazil’s unique overlapping social structures to reduce excess surgical births [40].

## Data and methods

This study quantifies the source of racial and ethnic disparities in CS rates in Brazil using multivariate logistic regression followed by a decomposition analysis using the Karlson-Holm-Breen (KHB) decomposition method [41]. The KHB method compares the coefficients between logistic regression models to quantify the variance between racial/ethnic groups explained by between-group differences in observable characteristics. We calculated the mediating effects of sociodemographic, pregnancy, and geographic factors as percentages of the total effect of race/ethnicity on the likelihood of a CS birth.

We used the 2019 national registry of live births (the latest year before the COVID-19 pandemic) published by the Brazil Ministry of Health’s Information System (SINASC in Portuguese). SINASC compiles and publishes demographic, socioeconomic, and medical information from the declaration of live births (*Documentário Nascidos Vivos* [DNV]), a document that healthcare providers issue for each birth. The SINASC dataset provides a broad range of information, including characteristics of mothers, pregnancies, and births [42].

We limited the analysis to hospital-based births by women aged 15–49, dropping 0.53% of births to women under 15 or over 49 years. We also excluded births outside the hospital, as none were CS births. Additionally, we dropped observations with missing values; 0.07% had a missing outcome variable (vaginal or CS birth), 3.5% of data had missing values on the mother’s race and ethnicity, and 4.55% had missing values on whether the mother received adequate prenatal care. We did not apply imputation techniques, including multiple imputation approaches, as we had no a priori knowledge of how missingness was generated and considered them missing at random. We expect that potential biases will be minimal due to the low percentage of missing values. Our analytical dataset comprised 90.9% of the SINSAC 2019 data (n = 2,567,039).

The binary outcome variable was whether the birth was via CS (“yes” = 1 and “no” = 0). The key independent variable was race and ethnicity using the current Brazilian Census categories (“*branca”* [Eurodescendant], “*preta”* [Afrodescendant], “*parda”* [Euro-afrodescendant or Euro-native descendant], “*amarela”* [Asian descent], and “*indígena”* [Brazilian native]). Although the categories are an imperfect measure of race and ethnicity in Brazil [36], they help report and track differences between groups across various health and social indicators [43]. The multivariate regression models also included sociodemographic, prenatal care, and pregnancy risk factors identified as predictors of CS in prior research [44]. Sociodemographic characteristics included the mother’s age, her education level (“less than high school [HS],” “HS completion,” and “postsecondary education”), and partner status (“unpartnered” and “partnered”). The models also included the Kotelchuck Index to indicate whether the mother received adequate prenatal care and the Robson classification of birth to account for pregnancy risk factors leading to CS. The Kotelchuck index, which has been linked to birth outcomes, assesses the quality of prenatal care using the number of consultations as a percentage of the expected consultations relative to gestational age [45]. The Robson classification is a system that categorizes pregnant women into ten major groups based on characteristics available at birth: parity, the onset of labor, gestational age, previous CS, fetal presentation, and number of fetuses [46]. Studies using SINASC data have found significant associations between CS and the Robson Classification [47,48].

We used a logistic regression model to examine the association between race and ethnicity and CS births while accounting for sociodemographic characteristics, prenatal care, and pregnancy risk factors. The regression model also included state-fixed effects to account for significant regional variation in CS rates within Brazil [49], and standard errors were clustered by municipality. The federative units of Brazil include 26 states and one federal district. The states are further divided into municipalities. In total, Brazil comprises 5,507 municipalities nested within states. The decomposition analysis builds upon the logistic regression model to assess the contributions of sociodemographic characteristics, prenatal care, pregnancy risk factors, and state of birth in examining racial and ethnic disparities in CS. Researchers used similar methodological approaches to study CS disparities in the United States [50]. We decided to use *Parda* (Euro-afro-descendant or Euro-native descendant) women as the reference category as the largest number of births were to *parda* women, and their CS rates were not at extremes (very high or very low).

## Results

### Descriptive characteristics

The plurality of births in 2019 Brazil were to *parda* women (57.4%). About 52.5% of births to *parda* women were via CS. *Branca* and *amarela* women had higher rates of CS at 66.8% and 58% respectively (Table 1). They were also more likely to be older, have more education, live with a partner, and have received adequate prenatal care. In contrast, *indígena* women were younger, less educated, unpartnered, and received less than adequate prenatal care compared to *parda* women. *Indígena* women were also the least likely to give CS births. While smaller in number, births to *preta* women had a similar profile to births among *parda* women. Regional differences exist, such that four states comprising Brazil’s Southeast region contribute over 40% of all births.

**Table 1:** Maternal characteristics of births in Brazil, 2019.

	All births	Parda	Branca	Preta	Amarela	Indígena	p-value
**Number of births**	2,567,039	1,448,537	924,985	164,612	12,025	16,880	
**Birth Mode (%)**							<0.001
Vaginal birth	42.6	47.5	33.2	49.7	42.0	69.7	
Cesarean (CS) birth	57.4	52.5	66.8	50.3	58.0	30.3	
**Median age of mother (years)**	27	26	29	27	29	24	<0.001
**Maternal education (%)**							<0.001
Less than high school	25.2	30.6	15.8	28.8	17.2	52.2	
High school completion	52.7	55.6	47.9	56.3	47.9	41.8	
Postsecondary education	22.0	13.8	36.3	14.9	34.9	6.0	
**Marital status (%)**							<0.001
Unpartnered	46.7	50.0	39.7	56.5	42.0	56.0	
Partnered	53.3	50.0	60.3	43.5	58.0	44.0	
**Prenatal care**^1^ **(%)**							<0.001
Received less than adequate care	18.4	21.4	12.6	23.1	15.6	37.0	
Received adequate care	81.6	78.6	87.4	76.9	84.4	63.0	
**Robson classification**^2^ **(%)**							<0.001
1. Nulliparous with a single cephalic pregnancy, ≥ 37 weeks gestation, spontaneous labor	17.6	19.1	15.4	16.0	17.1	17.3	
2. Nulliparous with a single cephalic pregnancy, ≥ 37 weeks gestation, induced labor or CS before labor	14.0	11.0	18.9	13.9	16.9	5.7	
3. Multiparous without a previous CS, single cephalic pregnancy, ≥ 37 weeks gestation, spontaneous labor	19.4	23.1	12.9	21.2	16.4	37.9	
4. Multiparous without a previous CS, single cephalic pregnancy, ≥ 37 weeks gestation induced labor or CS before labor	9.1	8.7	9.4	11.2	10.0	6.6	
5. Multiparous with at least one previous CS, single cephalic pregnancy, ≥ 37 weeks gestation	24.4	22.7	27.6	22.0	23.4	15.2	
6. Nulliparous with a single breech pregnancy	1.3	1.1	1.7	1.0	1.7	0.7	
7. Multiparous with a single breech pregnancy	1.9	1.8	2.0	1.8	1.8	1.9	
8. Multiple pregnancy	2.2	1.9	2.5	2.4	2.7	1.7	
9. Single pregnancy with a transverse or oblique lie	0.2	0.2	0.2	0.2	0.3	0.4	
10. Single cephalic pregnancy < 37 weeks gestation	8.9	9.1	8.5	9.2	8.6	11.4	
11. Incomplete data	1.1	1.3	0.8	0.9	1.1	1.3	

Tests of significance use the Pearson’s chi-squared test. Study is limited to hospital births to women aged 15–49. Data source: Brazil Ministry of Health’s Information System (SINASC)

^1^ Kotelchuck (1994).

^2^ Vogel et al. (2015).

### Regression analysis

Table 2 displays the results of the multivariate logistic regression model for the effects of race and ethnicity on the likelihood of CS, controlling for sociodemographic characteristics (age, educational level, and partner status), prenatal care and pregnancy risk factors, and geographic (state where the birth took place) factors. Compared to *parda* women, *branca* women were 34% more likely to have a CS birth after accounting for sociodemographic, health, and geographic covariates. In contrast, *preta, amarela, and indígena* women have lower risks of CS birth; *preta* women were 10% less likely, *amarela* women were 11% less likely, and *indígena* were women 45% less likely than *parda* women to have a CS birth.

**Table 2:** Logistics regression of likelihood of a CS birth on maternal characteristics in Brazil, 2019.

(N = 2,567,039)	Odds Ratio^1^	Coef.	Sig.	95% CI
**Race/Ethnicity**				
Parda	(reference)			
Branca	1.34	0.289	^***^	[0.266 – 0.313]
Preta	0.90	−0.102	^***^	[-0.128 – -0.077]
Amarela	0.89	−0.113	^***^	[-0.172 – -0.053]
Indígena	0.55	−0.590	^***^	[-0.685 – -0.496]
**Age of mother**	1.05	0.050	^***^	[0.049 – 0.052]
**Maternal education**				
Less than high school	(reference)			
High school completion	1.34	0.295	^***^	[0.282 – 0.308]
Postsecondary education	2.24	0.808	^***^	[0.773 – 0.844]
**Marital status**				
Unpartnered	(reference)			
Partnered	1.17	0.158	^***^	[0.131 – 0.185]
**Prenatal care** ^2^				
Received less than adequate care	(reference)			
Received adequate care	1.44	0.368	^***^	[0.347 – 0.389]
**Robson classification** ^3^				
1. Nulliparous with a single cephalic pregnancy, ≥ 37 weeks gestation, spontaneous labor	(reference)			
2. Nulliparous with a single cephalic pregnancy, ≥ 37 weeks gestation, induced labor, or CS before labor	2.98	1.093	^***^	[1.006 – 1.18]
3. Multiparous without a previous CS, single cephalic pregnancy, ≥ 37 weeks gestation, spontaneous labor	0.26	−1.349	^***^	[-1.37 – -1.328]
4. Multiparous without a previous CS, single cephalic pregnancy, ≥ 37 weeks gestation induced labor or CS before labor	1.00	−0.001		[-0.1 – 0.097]
5. Multiparous with at least one previous CS, single cephalic pregnancy, ≥ 37 weeks gestation	6.05	1.800	^***^	[1.756 – 1.845]
6. Nulliparous with a single breech pregnancy	14.70	2.688	^***^	[2.562 – 2.814]
7. Multiparous with a single breech pregnancy	8.52	2.143	^***^	[2.008 – 2.278]
8. Multiple pregnancy	6.06	1.802	^***^	[1.674 – 1.93]
9. Single pregnancy with a transverse or oblique lie	48.18	3.875	^***^	[3.633 – 4.117]
10. Single cephalic pregnancy < 37 weeks gestation	1.34	0.296	^***^	[0.215 – 0.378]
11. Incomplete data	1.94	0.665	^***^	[0.495 – 0.834]
**State Code & Name**				
(53) Distrito Federal	(reference)			
(11) Rondônia	2.34	0.850	^***^	[0.451 – 1.249]
(12) Acre	1.27	0.242	^***^	[0.138 – 0.347]
(13) Amazonas	1.24	0.216	^**^	[0.063 – 0.369]
(14) Roraima	0.79	−0.240	^***^	[-0.38 – -0.1]
(15) Pará	1.63	0.491	^***^	[0.373 – 0.608]
(16) Amapá	0.67	−0.404	^***^	[-0.48 – -0.327]
(17) Tocantins	1.55	0.439	^***^	[0.256 – 0.622]
(21) Maranhão	1.69	0.525	^***^	[0.442 – 0.608]
(22) Piaui	2.11	0.748	^***^	[0.635 – 0.861]
(23) Ceará	1.75	0.558	^***^	[0.473 – 0.643]
(24) Rio Grande do Norte	1.79	0.584	^***^	[0.342 – 0.826]
(25) Paraíba	1.98	0.684	^***^	[0.517 – 0.85]
(26) Pernambuco	1.25	0.226	^***^	[0.099 – 0.353]
(27) Alagoas	1.53	0.427	^***^	[0.339 – 0.514]
(28) Sergipe	0.76	−0.271	^***^	[-0.343 – -0.2]
(29) Bahia	1.09	0.083	^*^	[0.01 – 0.157]
(31) Minas Gerais	1.10	0.092		[-0.113 – 0.298]
(32) Espírito Santo	1.35	0.302	^***^	[0.187 – 0.417]
(33) Rio de Janeiro	1.32	0.278	^*^	[0.035 – 0.521]
(35) São Paulo	0.95	−0.047		[-0.219 – 0.125]
(41) Parana	1.11	0.105		[-0.017 – 0.228]
(42) Santa Catarina	0.84	−0.179	^**^	[-0.289 – -0.069]
(43) Rio Grande do Sul	1.10	0.093		[-0.083 – 0.27]
(50) Mato Grosso do Sul	1.84	0.612	^***^	[0.408 – 0.816]
(51) Mato Grosso	1.56	0.442	^**^	[0.133 – 0.75]
(52) Goías	2.15	0.766	^***^	[0.637 – 0.895]

* p < 0.05; ^**^ p < 0.01; ^**^ p < 0.001

^1^ Odds ratios are exponentiated coefficients to facilitate interpretation.

^2^ Kotelchuck (1994).

^3^ Vogel et al. (2015).

Multivariate logistic model accounts for clustering at the municipal level. Study is limited to hospital births to women aged 15–49.

Data Source: Brazil Ministry of Health’s Information System (SINASC).

### Decomposition analysis

Tables 3–6 display the key summary measures from the KHB decomposition, building on the multivariate regression analysis. We estimated the contributions of sociodemographic characteristics, prenatal care, pregnancy risk factors, and geographic factors to the race and ethnic disparity in CS births. In the decomposition results, percentages greater than 100 indicate that the mediators have a greater association with CS birth than race and ethnicity. Moreover, negative percentages indicate that the mediators are associated with CS birth in a direction opposite to the association of race and ethnicity with CS birth.

**Table 3:** Contribution of mother’s sociodemographic characteristics to race and ethnic disparities in likelihoods of CS in Brazil, 2019.

(reference: Parda)	Branca		Preta		Amarela		Indígena	
***A. Mediation by mother’s socioeconomic characteristics***								
Total disparity (A)^1^	0.544^***^		−0.071^***^		0.111^***^		−0.719^***^	
Mediation effect (B)^2^	0.255^***^		0.031		0.224^***^		−0.128^**^	
Confounding ratio	1.9		0.7		−1.0		1.2	
Confounding % (100^*^B/A)^3^	46.8		−43.9		201.2		17.9	
***B. Contribution of each factor to mediation and overall race and ethnic disparity***								
	**Branca**		**Preta**		**Amarela**		**Indígena**	
	% of mediation	% of disparity^4^	% of mediation	% of disparity^4^	% of mediation	% of disparity^4^	% of mediation	% of disparity^4^
Mother’s age	36.5	17.1	86.3	−37.8	39.8	80.0	39.1	7.0
High school completion (ref: < HS)	−12.5	−5.9	−9.8	4.3	−12.5	−25.2	29.7	5.3
Postsecondary schooling (ref: < HS)	69.0	32.3	38.6	−16.9	67.7	136.3	33.9	6.1
Partnered (ref: unpartnered)	7.0	3.3	−15.1	6.6	5.1	10.2	−2.7	−0.5
**Sum**	100.0	46.8	100.0	−43.9	100.0	201.2	100.0	17.9

* p < 0.05; ^**^ p < 0.01; ^**^ p < 0.001

^1^ Total disparity is the effect of race and ethnicity without mediators and after controlling for concomitants (controls).

^2^ Negative mediation effects indicate that the race and ethnic group’s characteristics are negatively associated with the likelihood of CS.

^3^ Confounding percentage is the mediation effect as a percentage of the total disparity. Negative confounding percentages indicate mediation effects that run counter to the total disparity. Percentages greater than 100 indicate that the mediators have a greater association with CS birth than race and ethnicity.

^4^ Sums to the confounding percentage. Decomposition analysis is based on the multivariate logit model on likelihood of a CS (Table 2). The model controls for prenatal care, Robson Classification and birth state, and accounts for clustering by municipality.

Data Source: Brazil Ministry of Health’s Information System (SINASC).

### Mediation by sociodemographic factors (Table 3)

Regression results (Table 2) showed that births among older mothers had a higher likelihood of being a CS birth; the risk of CS increased by 5% for each year increase in the mother’s age. Partnered women were more likely than unpartnered women to give a CS birth (OR: 1.17), and compared to women without a high school degree, women with a high school degree or postsecondary education had higher rates of CS births (OR: 1.34 and 2.24, respectively). *Branca* and *amarela* women had sociodemographic profiles that predicted a greater likelihood of CS. They were older on average than *parda* women, more likely to be partnered, and had greater educational attainment (Table 1). Collectively, sociodemographic factors accounted for 46.8% of the CS disparity between *branca* and *parda* women, of which differences in age and postsecondary education were the largest contributors (Table 3). Age and postsecondary education were also prominent factors for *amarela* women, and the mediating impact more than twice exceeded the observed disparity between *amarela* and *parda* women (201.2%). In contrast, *indígena* women had sociodemographic profiles associated with lower CS rates, accounting for 17.9% of their lower CS rates compared to *parda* women. Sociodemographic factors were significant mediators for CS differences for *branca*, *amarela*, and *indígena* women. *Preta* women, however, had sociodemographic profiles similar to *parda* women; age, education, and partnership status did not significantly mediate *preta* women’s lower CS rates (OR: 0.90 ref: *parda*). In summary, higher maternal age and educational attainment among *branca* and *amarela* significantly mediated their higher CS rates compared to *parda* women. Age and education also mediated the lower CS rates among *indígena* women, but sociodemographic differences had little overall effect on *preta* women’s CS rates.

### Mediation by access to prenatal care (Table 4)

Women who received adequate or more than adequate care were 44% more likely to get a CS than women who received less than adequate care (Table 2). Table 4 shows how much prenatal care explained racial and ethnic differences in CS. More *branca* women had adequate prenatal care than *parda* women, and this difference in access explained 2.8% of the CS disparity. While *amarela* women had similar high access to prenatal care, it had little effect on the CS disparity (Table 4). *Preta* and *indígena* women had lower access to prenatal care than *parda* women, which explained about 10.6% and 4.1% of their lower CS rates, respectively.

**Table 4:** Contribution of access to prenatal care to race and ethnic disparities in likelihoods of CS in Brazil, 2019.

(reference: Parda)	Branca	Preta	Amarela	Indígena
**Mediation by mother’s receipt of adequate prenatal care**				
Total disparity (A) ^1^	0.298^***^	−0.114^***^	−0.112^***^	−0.616^***^
Mediation effect (B) ^2^	0.008^*^	−0.012^**^	0.000	−0.025^***^
Confounding % (100^*^B/A) ^3^	2.8	10.6	−0.4	4.1

* p < 0.05; ^**^ p < 0.01; ^**^ p < 0.001

^1^ Total disparity is the effect of race and ethnicity without mediators and after controlling for concomitants (controls).

^2^ Negative mediation effects indicate that the race and ethnic group’s characteristics are negatively associated with the likelihood of CS.

^3^ Confounding percentage is the mediation effect as a percentage of the total disparity. Negative confounding percentages indicate mediation effects that run counter to the total disparity. Percentages greater than 100 indicate that the mediators have a greater association with CS birth than race and ethnicity.

Data Source: Brazil Ministry of Health’s Information System (SINASC).

### Mediation by pregnancy risk classification (Table 5)

The reference category Robson group 1 covers nulliparous births with a single fetus in ≥37 weeks of gestation and spontaneous labor. Robson groups 5–10 cover births with classic medical indications for a CS, including a history of CS, breech pregnancy, multiple fetuses, transverse or oblique fetal position, and premature gestation of <37 weeks. The estimated odds of CS in Robson groups 5–10 are large. For example, birth with a transverse or oblique fetal position (Robson 9) was 48 times more likely to be via CS than the reference group. Whereas Robson groups 1–2 cover births with a single fetus, without a history of CS, and with ≥37 weeks of gestation. Among these, Robson group 2 was 198% more likely, and Robson group 3 was 74% less likely than the reference group to have a CS. Robson group 4 was just as likely to have a CS birth as the reference group.

**Table 5:** Contribution of mother’s medical risk factors to race and ethnic disparities in likelihoods of CS in Brazil, 2019.

(reference: Parda)	Branca		Preta		Amarela		Indígena	
***A. Mediation by mother’s medical risk***								
Total disparity (A)^1^	0.428^***^		−0.131^***^		−0.076^*^		−0.854^***^	
Mediation effect (B)^2^	0.131^***^		−0.018		0.036		−0.239^***^	
Confounding % (100^*^B/A)^3^	30.6		13.4		−47.4		28.0	
***B. Contribution of each factor to mediation and overall race and ethnic disparity***								
	**Branca**		**Preta**		**Amarela**		**Indígena**	
	% of mediation	% of disparity^4^	% of mediation	% of disparity^4^	% of mediation	% of disparity^4^	% of mediation	% of disparity^4^
1. Nulliparous with a single cephalic pregnancy, ≥ 37 weeks gestation, spontaneous labor	(reference)							
2. Nulliparous with a single cephalic pregnancy, ≥ 37 weeks gestation, induced labor or CS before labor	21.0	6.4	−41.7	−5.6	61.8	−29.3	6.9	1.9
3. Multiparous without a previous CS, single cephalic pregnancy, ≥ 37 weeks gestation, spontaneous labor	44.6	13.6	0.4	0.1	79.2	−37.5	52.0	14.6
4. Multiparous without a previous CS, single cephalic pregnancy, ≥ 37 weeks gestation induced labor or CS before labor	0.2	0.1	0.6	0.1	0.0	0.0	−0.1	0.0
5. Multiparous with at least one previous CS, single cephalic pregnancy, ≥ 37 weeks gestation	23.7	7.3	149.5	20.0	−91.9	43.6	49.9	14.0
6. Nulliparous with a single breech pregnancy	7.5	2.3	14.3	1.9	26.2	−12.4	0.8	0.2
7. Multiparous with a single breech pregnancy	1.2	0.4	1.3	0.2	−5.7	2.7	−2.8	−0.8
8. Multiple pregnancy	1.5	0.5	−27.8	−3.7	17.4	−8.3	−0.6	−0.2
9. Single pregnancy with a transverse or oblique lie	−0.1	0.0	5.5	0.7	9.3	−4.4	−3.4	−0.9
10. Single cephalic pregnancy < 37 weeks gestation	−0.2	−0.1	−3.8	−0.5	−0.6	0.3	−1.7	−0.5
11. Incomplete data	0.7	0.2	1.7	0.2	4.3	−2.0	−1.1	−0.3
**Sum**	100.0	30.6	100.0	13.4	100.0	−47.4	100.0	28.0

* p < 0.05; ^**^ p < 0.01; ^**^ p < 0.001

^1^ Total disparity is the effect of race and ethnicity without mediators and after controlling for concomitants (controls).

^2^ Negative mediation effects indicate that the race and ethnic group’s characteristics are negatively associated with the likelihood of CS.

^3^ Confounding percentage is the mediation effect as a percentage of the total disparity. Negative confounding percentages indicate mediation effects that run counter to the total disparity. Percentages greater than 100 indicate that the mediators have a greater association with CS birth than race and ethnicity.

^4^ Sums to the confounding percentage. Decomposition analysis is based on the multivariate logit model on likelihood of a CS (Table 2). The model controls for sociodemographic factors, prenatal care and birth state, and accounts for clustering by municipality.

Data Source: Brazil Ministry of Health’s Information System (SINASC).

The results in Table 5 show that after controlling for other covariates, pregnancy risk classification significantly contributed to the CS disparity for *branca* women (30.6%) and *indígena* women (28%). For both racial and ethnic groups, most of the pregnancy risk mediation effect comes from Robson Group 3 (44.6% for *branca* women and 52% for *indígena* women) and Robson Group 5 (23.7% for *branca* women and 49.9% for *indígena* women).

### Mediation by state of birth (Table 6)

**Fig 1:**
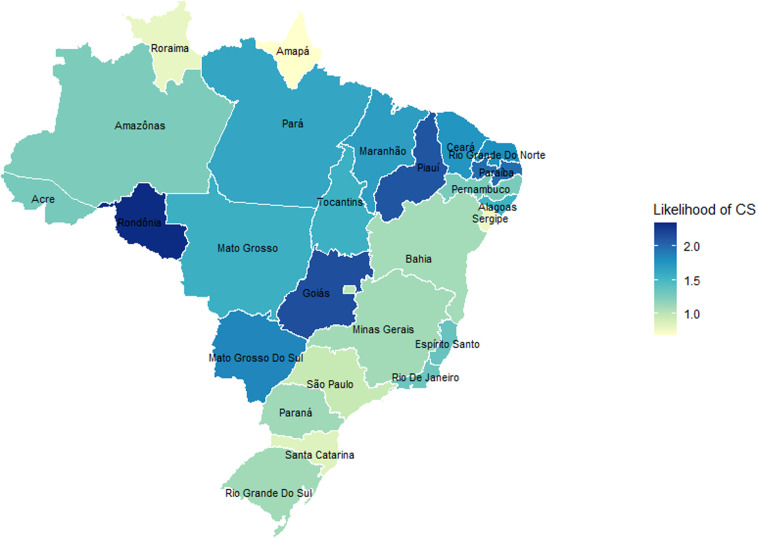
Odds of CS births by state in Brazil in 2019 after controlling for individual-level characteristics.

**Fig 2:**
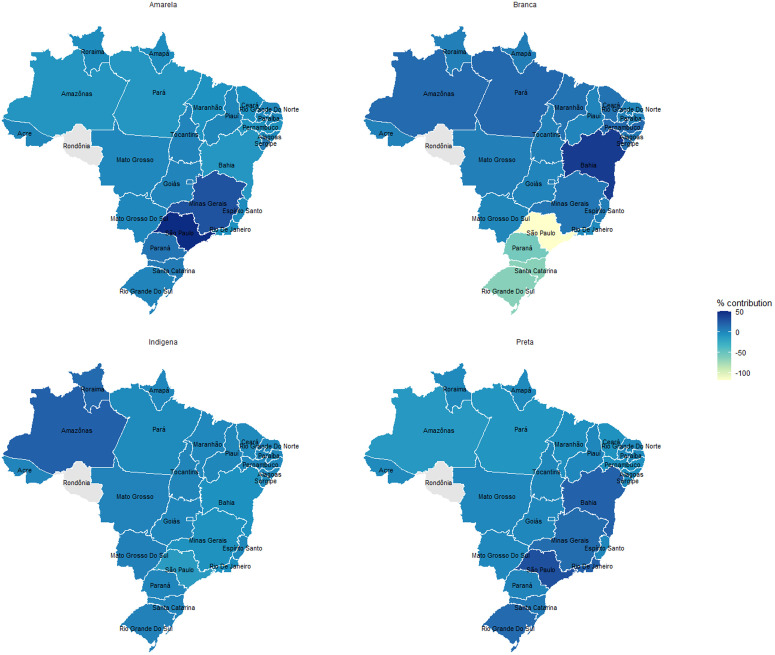
Mediating contribution of state of birth to race and ethnic disparities in CS in Brazil in 2019.

Fig 1 maps the likelihood of birth by CS in Brazilian states after controlling for individual-level sociodemographic, prenatal, and pregnancy risk factors. All states except Minas Gerais, São Paulo, Paraná, and Rio Grande do Sul significantly differed from the reference state, Distrito Federal. The state of birth significantly contributes to disparities in CS births for *branca* women (−131.6%), *amarela* women (42.4%), and *preta women* (53%) compared to *parda* women (Table 6). Most births among *branca* women were concentrated in São Paulo (33.4%), Paraná (11.7%), and Rio Grande do Sul (11.4%). These state profiles also had prominent contributions to the mediation effect for *branca* women, albeit in a counter direction to the effect of race and ethnicity, as indicated by the negative confounding percentage (Fig 2). In other words, *branca* women, whose CS rates were significantly higher than *parda* women, tended to give birth in places that had lower CS risk. The majority of births by *amarela* and *preta* women also occurred in eastern states near the coast (São Paulo and Minas Gerais for *amarela* women; São Paulo, Rio de Janeiro, and Bahia for *preta* women), which significantly contributed to their lower CS compared to *parda* women (Fig 2). On the other hand, *indígena* women are more concentrated in Amazonas and Roraima, a large and sparsely populated region in the northwest where CS rates are far below national levels. Unlike *amarela* and *preta* women, however, *indígena* women’s location contributes little to their relatively low CS rates. In sum, *branca*, *amarela*, and *preta* women were concentrated in states with relatively lower CS rates, which contributed to the racial and ethnic disparity compared to *parda* women.

**Table 6:** Contribution of birth state to race and ethnic disparities in likelihoods of CS in Brazil, 2019.

(reference: Parda)	Branca	Preta	Amarela	Indígena
**Mediation by mother’s birth state**				
Total disparity (A)^1^	0.125^***^	−0.218^***^	−0.196^***^	−0.618^***^
Mediation effect (B)^2^	−0.164^***^	−0.115^**^	−0.083^*^	−0.028
Confounding % (100^*^B/A)^3^	−131.6	53.0	42.4	4.5

* p < 0.05; ^**^ p < 0.01; ^**^ p < 0.001

^1^ Total disparity is the effect of race and ethnicity without mediators and after controlling for concomitants (controls).

^2^ Negative mediation effects indicate that the race and ethnic group’s characteristics are negatively associated with the likelihood of CS.

^3^ Confounding percentage is the mediation effect as a percentage of the total disparity. Negative confounding percentages indicate mediation effects that run counter to the total disparity. Percentages greater than 100 indicate that the mediators have a greater association with CS birth than race and ethnicity.

Data Source: Brazil Ministry of Health’s Information System (SINASC).

## Discussion

Our analysis of Brazil’s national birth registry data examined sociodemographic, geographic, and pregnancy risk factors as mediators for the large disparities in CS between different race and ethnic groups. The greater risk of CS among *branca* women than other groups persisted even after controlling for covariates.

Decomposition analyses showed that sociodemographic factors were the largest contributors to the *branca-parda* and *amarela-parda* CS gaps, and older maternal age and greater educational attainment were the predominant factors in elevated CS rates among *branca* and *amarela* women.

Inequalities in educational opportunity by race and ethnicity in Brazil have persisted beyond waves of affirmative action policies since the early 2000s. While the racial inequality in the likelihood of entering high school has abated, there is an increase in non-white disadvantage in completing high school [51]. Black women tend to have lower educational attainment, which correlates with income and other measures of socioeconomic resources [52]. Lower educational attainment is associated with lower quality of maternal care, but does not completely explain racial/ethnic differences; women with darker skin color are more likely to receive inadequate or delayed care even when controlling for education [53].

Yet, while socioeconomic advantage predicts fewer CS in most populations [44], the link between education and CS in Brazil ran counter to studies conducted in other racialized contexts, such as the United States [54]. Our analysis showed that greater educational attainment among *branca* and *amarela* women was a factor that contributed to their greater CS rates, and their older maternal ages further expanded the gap compared to *parda* women.

Our results also showed that prenatal care’s role in racial and ethnic CS disparities in Brazil differed from studies in other contexts. Less-than-adequate prenatal care contributed to heightened CS rates among Black mothers in the United States [54] and among women of lower socioeconomic status in France [55]. Racial and ethnic disparities in prenatal care did not emerge as a significant mediator in this study, and the results suggest that other socioeconomic and geographic factors had stronger associations with CS disparities in Brazil. This finding is consistent with other research from Brazil that shows women of higher social class with greater access to prenatal care also have higher rates of CS births [56,57]. Disparate CS rates stemming from social inequities directly translate to unequal biomedical risk that increases women’s risk of CS in subsequent pregnancies [58].

History of prior CS delivery in a prior pregnancy was a notable source of higher CS rates among *branca* women. Conversely, *indígena* women’s low CS rates in a prior pregnancy translated to a lower pregnancy risk profile that contributed to their overall low CS rate compared to *parda* women. Our findings demonstrate the reinforcing cycle of racial and ethnic CS disparity across Brazilian women’s repeated pregnancies [59]. The analysis suggests that existing racial and ethnic differences in CS risk may reinforce disparities across Brazilian women’s repeated pregnancies [59].

While the decomposition analysis provided insight into possible underlying factors that contribute to racial/ethnic disparities in CS rates, unobserved differences may also drive these disparities. Among unobserved variables, regional differences in healthcare systems and access are likely contributors. The confluence of unobserved socioeconomic differences may further complicate regional variations in CS rates observed in our analysis. Broadly, socioeconomic advantage is concentrated in the South, Southeast, and Central-West regions of the country, where our models uncovered relatively lower region-level rates of CS after controlling for individual-level variables. Contradictory relationships between individual-level advantage and region-level advantage and CS suggest that regional concentration of socioeconomic advantage may reduce CS through healthcare infrastructure and access. Possible differences in maternal healthcare infrastructure between regions that could drive CS rates may include a lack of proper facilities or qualified personnel for various modes of delivery [60]. Thus, the uneven racial and ethnic distribution of mothers across Brazil’s regions shaped the patterns of CS disparities at the national level, independent of women’s individual CS risk, and regional health policy and resource allocation may present opportunities for interventions to reduce CS rates. Specific mechanisms that produce geographic differences in CS merit further examination.

Our analysis also did not include women who gave birth outside hospitals and thus overestimated actual CS rates; non-hospital births are almost universally vaginal births. Overestimation may be particularly high for *indígena* women who are more likely to give birth outside hospitals and have home births in indigenous villages [60]. Thus, actual CS rates among all *indígena* women were likely lower than those of other racial and ethnic groups, and our results likely underestimated between-group disparities. If having greater socioeconomic resources (i.e., education) and more prenatal care are correlated to a hospital birth, the contribution of those characteristics to the disparity between *branca* and *indígena* women may be understated in the decomposition results. Yet, even with the likely overestimated CS rates among *indígena* women in Brazil, their relatively lower rates are markedly different from those in the United States, where American Indian and Alaska Native women undergo CS births more frequently than white women [61].

## Conclusion

Brazil’s CS epidemic has drawn the attention of numerous stakeholders, including researchers, national and global policymakers, advocacy groups, and journalistic outlets. Social movement initiatives and public policies have used campaigns and social mobilization for the humanization of childbirth to reduce unnecessary CS [62,63]. Moreover, based on the debates around CS, scholars have proposed the inclusion of “obstetric culture” as a risk factor for surgical births [44].

This study highlights the potential role that Brazil’s decentralized and fragmented healthcare system may play in creating disparities in CS rates, as well as their overall high prevalence. The public and private sectors are closely integrated (SUS partially finances private care), but the use of privatized care is unequal by socioeconomic status and geographic region [64]. Private healthcare providers are concentrated in the wealthy southeast region, and people with higher incomes and greater occupational status are more likely to have insurance plans that allow them greater access to private providers and facilities [49]. Healthcare delivery in the private sector is often centered on patient demand rather than population-based promotion strategies, and the fee-for-service payment structures incentivize supplier-induced procedures [26]. In such a healthcare delivery environment, CS rates would be elevated among women with advantaged socioeconomic characteristics—a connection that is reflected in our findings.

Brazil’s decentralized healthcare system adds challenges to reducing medically unnecessary CS procedures. More than 5,500 municipalities nested in five geographic regions co-finance, manage, and deliver their localities’ healthcare services [65]. While national policies manage select high-cost care (e.g., organ transplants and cardiac surgery), systematic monitoring and regulatory mechanisms are less developed for CS deliveries [26]. Stronger integration between public and private maternal health services at all levels of care—primary, secondary, and tertiary—can set the stage to introduce care standards that improve the health of mothers and their newborns. Efforts to reduce CS disparities must also address the broader systemic inequalities that affect minoritized racial and ethnic populations in Brazil. Despite evidence of large and persistent disparities in CS and maternal health by race and ethnicity in Brazil, public health efforts to reduce excess CS place little emphasis on acknowledging broader social and structural factors that produce these differences [15,29]. For example, the majority of Brazil’s public health response to high CS rates centers on providing education on birth processes and risks of CS without considering the contexts in which women make decisions and give birth [29]. A national policy *Rede Cegonha* (Stork Network) promotes low-intervention, evidence-based care that prioritizes vaginal delivery over CS when appropriate. However, *Rede Cegonha* contains no language about race and ethnicity and their interactions with the broader social and healthcare service environments in the policy’s planning and implementation [15]. Interventions that solely focus on educating women about CS show limited impact; white and Black women showed similar knowledge about the risks of CS, yet racial and ethnic differences in CS rates persist [66]. Findings from this study indicate that efforts to reduce CS births in Brazil should recognize racial and ethnic groups as independent axes of inequality and design policies and interventions that target the multilevel characteristics affecting women’s risk of CS births.
